# Rheumatoid arthritis is independently associated with metabolic Dysfunction-Associated steatotic liver disease: evidence from the paracelsus 10,000 Population-Based cohort study

**DOI:** 10.1007/s00296-025-06002-2

**Published:** 2025-10-06

**Authors:** Mathias Ausserwinkler, Axel J. Hueber, Sophie Gensluckner, Bernhard Paulweber, Eugen Trinka, Patrick Langthaler, Christian Datz, Andreas Voelkerer, Franz Singhartinger, Bernhard Iglseder, Maria Flamm, Elmar Aigner, Bernhard Wernly

**Affiliations:** 1 Department of Internal Medicine, Elisabethinen Hospital Klagenfurt, Klagenfurt, Austria; 2 First Department of Medicine, Landeskrankenhaus—Uniklinikum Paracelsus Medical University Salzburg, Salzburg, Austria; 3 Division of Rheumatology, Klinikum Nuernberg, Paracelsus Medical University, Nürnberg, Germany; 4 Department of Neurology, Neurointensive Care and Neurorehabilitation, Member of the European Reference Network EpiCARE, Christian Doppler University Hospital, Centre for Cognitive Neuroscience, Paracelsus Medical University Salzburg, Nürnberg, Austria; 5 Neuroscience Institute, Christian Doppler University Hospital, Centre for Cognitive Neuroscience, Paracelsus Medical University Salzburg, Nürnberg, Austria; 6 Department of Internal Medicine, General Hospital Oberndorf, Teaching Hospital of the Paracelsus Medical University Salzburg, Nürnberg, Austria; 7 Department of General, Visceral and Thoracic Surgery, Paracelsus Medical University/Salzburger Landeskliniken (SALK), Salzburg, Austria; 8 Department of Geriatric Medicine, Christian Doppler University Hospital, Paracelsus Medical University Salzburg, Nürnberg, Austria; 9 Institute of General Practice, Family Medicine and Preventive Medicine, Center for Public Health and Healthcare Research, Paracelsus Medical University Salzburg, Nürnberg, Austria

**Keywords:** Rheumatoid arthritis, Metabolic dysfunction-associated steatotic liver disease, Fatty liver, Fibrosis, Cohort studies, Risk factors

## Abstract

**Background:**

Rheumatoid arthritis (RA) is associated with systemic inflammation and increased risk of cardiovascular and metabolic comorbidities. The relationship between RA and metabolic dysfunction-associated steatotic liver disease (MASLD) has not been established in population-based studies.

**Methods:**

We conducted a cross-sectional analysis of 6638 participants from the population-based Paracelsus 10,000 cohort in Austria, including 187 individuals with physician-diagnosed RA meeting ACR/EULAR classification criteria. MASLD was defined using the Fatty Liver Index (≥ 60) combined with cardiometabolic risk factors according to 2024 EASL guidelines. We used Poisson regression models with sequential adjustment for demographic factors, metabolic syndrome, lifestyle factors, NSAID use, and cardiovascular risk (SCORE2). Liver fibrosis risk was assessed using the Fibrosis-4 Index (FIB-4).

**Results:**

MASLD prevalence was higher in RA patients than controls (41.2% vs. 28.5%, *P* < 0.001). In sequential regression models, the association between RA and MASLD persisted after adjustment for demographics (IRR, 1.55; 95% CI 1.33–1.82), metabolic and lifestyle factors (IRR, 1.20; 95% CI 1.03–1.40), and cardiovascular risk factors (IRR, 1.35; 95% CI 1.14–1.60; *P* < 0.001). In addition, RA patients showed elevated liver fibrosis markers (median FIB-4: 1.21 vs. 1.08; *P* < 0.001).

**Conclusions:**

In this population-based cohort, RA was independently associated with a 35% increased risk of MASLD and elevated liver fibrosis markers. These findings suggest that systematic liver assessment should be considered in the routine care of RA patients.

## Introduction

RA is a chronic systemic autoimmune disease characterized by persistent synovial inflammation, primarily affecting joints and impacting approximately 1% of the global population​ [1]. Beyond joint inflammation, RA is associated with numerous extra-articular complications and comorbidities, including cardiovascular diseases, metabolic syndrome and liver dysfunction [2–4]. Despite the availability of various conventional and biological disease-modifying anti-rheumatic drugs the disease can remain difficult to control and typically leads to a lifelong treatment.

MASLD, formerly known as Non-Alcoholic Fatty Liver Disease (NAFLD) affects up to 30% of the adult population in Western countries, posing a significant global health challenge [5, 6]. It is characterized by the accumulation of fat in the liver along with metabolic risk factors such as obesity, type 2 diabetes or dyslipidemia. MASLD is diagnosed when these criteria are met and alcohol consumption remains below 20 g per day for women and 30 g per day for men, ruling out significant alcohol-related liver damage as a primary cause [7]. Approximately 5% of MASLD patients advance to more serious conditions like steatohepatitis, cirrhosis or hepatocellular carcinoma [5]. This makes MASLD a widespread condition which is associated with significant clinical and economic burdens [6, 8]. Furthermore, MASLD is linked to increased risks of cardiometabolic diseases, extrahepatic malignancies, diabetes and respiratory conditions [9].

The relationship between RA and liver disease is not recognized. Chronic inflammation in RA, as well as the effects of medications like methotrexate and NSAIDS, may contribute to liver injury. Despite this, there is limited research explicitly examining the link between RA and liver disease [10, 11].

The Paracelsus 10,000 study is a large, population-based cohort designed to investigate various health outcomes across a broad range of participants [12]. With over 10,000 individuals enrolled, it provides extensive data on both metabolic conditions and autoimmune diseases like RA. This makes it an excellent resource for exploring the potential correlations between MASLD and RA, offering insights into how chronic inflammation and metabolic factors interact in these patients [12–16].

To date, no large population-based study has specifically investigated the association between RA and MASLD. This study aims to fill that gap by analyzing data from a well-characterized Austrian cohort.

## Methods

### Study population

We conducted a cross-sectional analysis using data from the Paracelsus 10,000 cohort, a population-based observational study in Salzburg, Austria. Between April 2013 and March 2020, participants aged 40–77 years were randomly selected from the Austrian national registry. Of 56,600 invitations sent, 10,044 individuals participated.

For this analysis, we included 6,638 participants with complete data on liver function, cardiometabolic parameters and RA status. We excluded individuals with other chronic autoimmune diseases, missing laboratory values, or alcohol consumption above MASLD thresholds (> 20 g/day for women, > 30 g/day for men). As this was a secondary analysis of the existing Paracelsus 10,000 cohort, no formal sample size calculation was performed a priori. Our study had sufficient power (> 90%) to detect the observed 12% difference in MASLD prevalence between groups and could detect differences as small as 10.2% with 80% power at α = 0.05.” This explains both our methodological approach and demonstrates adequate statistical power.

### Definitions

RA was diagnosed by rheumatologists or internists using the 2010 ACR/EULAR classification criteria, confirmed through medical record review and interviews. MASLD was defined as FLI ≥ 60 plus at least one cardiometabolic risk factor (BMI ≥ 25 kg/m², type 2 diabetes, hypertension, dyslipidemia, or metabolic dysregulation), according to 2024 EASL-EASD-EASO guidelines. FLI incorporates BMI, waist circumference, triglycerides, and gamma-glutamyl transferase levels.

Liver fibrosis risk was assessed using the FIB-4 index, with participants categorized as low risk (< 1.3), intermediate risk (1.3–2.67), or high risk (> 2.67). Cardiovascular risk was evaluated using SCORE2 (ages 40–69) or SCORE2-OP (≥ 70 years).

NSAID use was assessed through structured interviews during the baseline examination, focusing on regular use within the preceding three months. This timeframe was chosen because NSAIDs can affect liver enzymes and inflammatory markers within this period, potentially confounding the relationship between RA and liver outcomes. We included NSAID adjustment in Model IV because these medications are commonly used in RA management and may contribute to liver enzyme abnormalities.

### Statistical analysis

All analyses were performed using Stata version 19.5. Continuous variables are presented as median (interquartile range) and categorical variables as frequencies (percentages). Group comparisons used Mann-Whitney U tests for non-normal continuous variables and chi-square tests for categorical variables.

We used Poisson regression to estimate incidence rate ratios (IRR) with 95% confidence intervals, as this approach is more appropriate than logistic regression for cross-sectional studies with common outcomes. Five sequential models were constructed: (1) unadjusted, (2) adjusted for age and sex, (3) additionally adjusted for metabolic syndrome, smoking, and income, (4) further adjusted for NSAID use, and (5) finally adjusted for cardiovascular risk scores.

Subgroup analyses examined associations stratified by sex, age (≤ 55 vs. >55 years), and metabolic syndrome status. Statistical significance was set at *P* < 0.05.

The study was approved by the Ethics Committee of the Province of Salzburg, Austria (approval no. 415-E/1521/3-2012, approval date 12 June 2012). The approval covers the Paracelsus 10,000 cohort study and subsequent secondary analyses. The study was conducted in accordance with the Declaration of Helsinki and Austrian regulatory requirements. All participants provided written informed consent prior to enrolment.

## Results

### Baseline characteristics

This study included 6,638 participants, of whom 187 (3%) were diagnosed with RA. Participants with RA were older (median age 58 vs. 54 years), more frequently female (69% vs. 54%), and had higher levels of systemic inflammation (hs-CRP: 0.14 vs. 0.11 mg/L, *p* = 0.010) compared to controls. RA patients had a significantly higher prevalence of metabolic syndrome (28% vs. 15%, *p* < 0.001) and obesity, but similar liver enzyme levels (AST and gamma-GT). Educational attainment was lower among RA participants, with fewer having higher education (16% vs. 24%, *p* < 0.001). Complete baseline characteristics are shown in Table 1.

### Primary outcome: MASLD prevalence and association

**Fig. 1:**
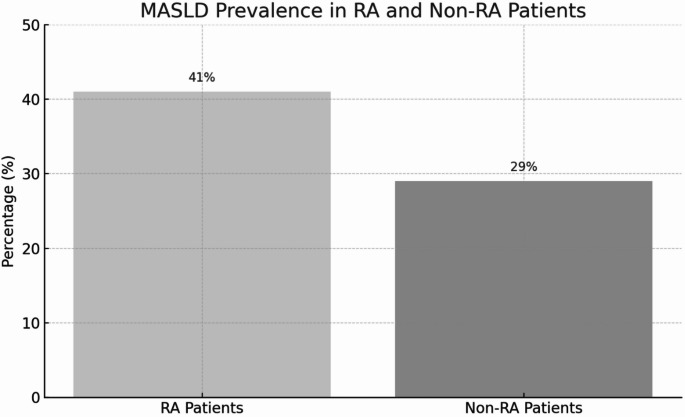
Prevalence of Metabolic Dysfunction-Associated Steatotic Liver Disease in Patients with and without Rheumatoid Arthritis. The prevalence of MASLD was significantly higher among patients with rheumatoid arthritis (RA) than among those without RA (41% vs. 29%, *P* < 0.001). MASLD was defined as a Fatty Liver Index of 60 or higher combined with at least one cardiometabolic risk factor, in accordance with 2024 EASL-EASD-EASO guidelines. The analysis included 187 participants with RA and 6,451 controls from the population-based Paracelsus 10,000 cohort

MASLD prevalence was higher among RA patients compared to controls [41% (77 individuals) vs. 29% (1842 individuals), *p* < 0.001] (Fig. 1). In sequential Poisson regression analyses, RA remained independently associated with MASLD across all adjustment models. The unadjusted association showed an IRR of 1.44 (95% CI 1.21–1.72, *p* < 0.001). After adjusting for age and sex, the association strengthened (IRR: 1.55, 95% CI 1.33–1.82, *p* < 0.001). Further adjustment for metabolic syndrome, smoking, and income attenuated but maintained the association (IRR: 1.20, 95% CI 1.03–1.40, *p* = 0.019). The addition of NSAID use reduced the association to borderline significance (IRR: 1.15, 95% CI 0.99–1.35, *p* = 0.076). In the final model incorporating cardiovascular risk scores (SCORE2), RA remained significantly associated with MASLD (IRR: 1.35, 95% CI 1.14–1.60, *p* < 0.001) - (Table 2).

### Secondary outcomes: liver fibrosis risk

**Fig. 2:**
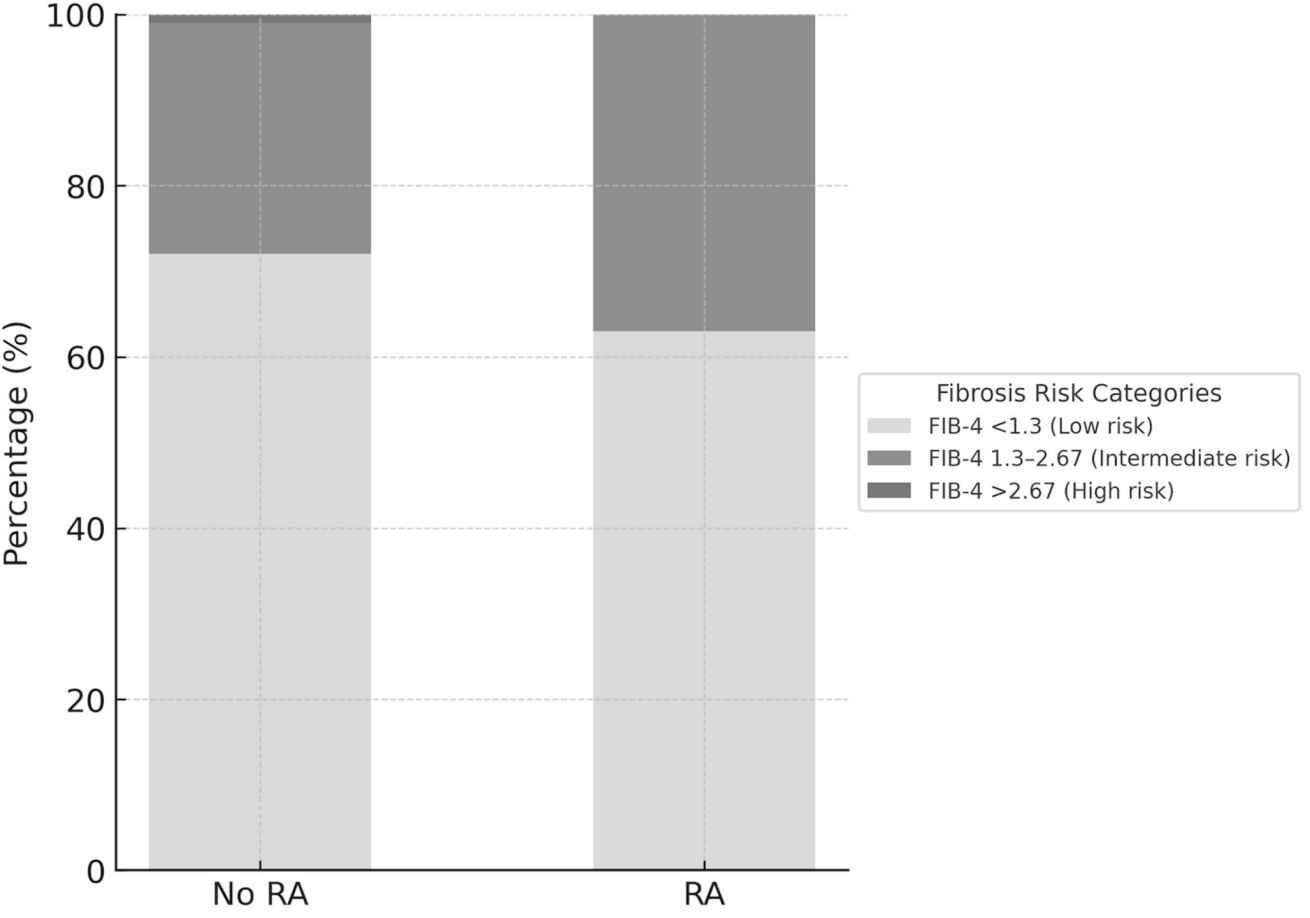
Distribution of Liver Fibrosis Risk Categories According to FIB-4 Index in Patients with and without Rheumatoid Arthritis. Patients with rheumatoid arthritis (RA) had a higher proportion of intermediate fibrosis risk compared with controls. Among participants with RA, 63% were classified as low risk (FIB-4 < 1.3), 37% as intermediate risk (FIB-4 1.3–2.67), and 0% as high risk (FIB-4 > 2.67). Among participants without RA, 72% were classified as low risk, 27% as intermediate risk, and 1% as high risk (*P* = 0.01). The FIB-4 index incorporates age, aspartate aminotransferase, alanine aminotransferase, and platelet count to assess liver fibrosis risk. Data are from 187 participants with RA and 6451 controls

RA patients showed elevated FIB-4 scores compared to controls [median: 1.21 (IQR 0.99–1.50) vs. 1.08 (IQR: 0.87–1.34), *p* < 0.001]. Using established FIB-4 cut-offs, fewer RA patients were classified as low risk (< 1.30: 63% vs. 72%) while more were in the intermediate risk category (1.30–2.67: 37% vs. 27%). No RA patients were classified as high risk (> 2.67) compared to 1% of controls (Fig. 2). However, after adjustment for demographic and clinical factors, the association between RA and elevated FIB-4 (> 1.30) was no longer statistically significant across models II-V (all *p* > 0.05).

### Subgroup analyses

In stratified analyses, the association between RA and MASLD was consistent across sex (females: IRR 1.64, 95% CI 1.23–2.19; males: IRR 1.68, 95% CI 1.42–2.00, both *p* < 0.01) but varied by age, with significant associations only in participants older than 55 years (IRR: 1.42, 95% CI 1.17–1.73, *p* < 0.001). Among participants without metabolic syndrome, RA remained associated with MASLD (IRR: 1.36, 95% CI 1.01–1.84, *p* = 0.046), while no association was observed in those with pre-existing metabolic syndrome (IRR: 0.98, 95% CI 0.86–1.11, *p* = 0.719).

## Discussion

This population-based cohort study demonstrates that RA is associated with an increased prevalence of MASLD. Notably, this association remained robust after adjustment for age, sex and metabolic risk factors, underscoring MASLD as a relevant comorbidity in RA beyond classical determinants. To our knowledge, this is the first large, population-based study specifically addressing this association, thereby providing robust epidemiological evidence for this previously underexplored link.

Recent research indicates that the updated MASLD terminology not only reflects a shift in nomenclature but also underscores its strong links to systemic metabolic and inflammatory disorders [17, 18]. In line with this, a meta-analysis showed that autoimmune rheumatic diseases carry an increased risk of MASLD, supporting the role of systemic inflammation in liver disease development [19]. Evidence further suggests that MASLD in chronic inflammatory conditions may share common mechanistic pathways, reinforcing the importance of systematic liver assessment in these patients [20, 21]. Prior studies have reported a high burden of hepatic abnormalities in RA, often related to systemic inflammation and treatment-related hepatotoxicity [22].

Our findings extend these observations by identifying MASLD as the predominant hepatic phenotype in a large population-based RA cohort. These results are consistent with recent data demonstrating a high prevalence of comorbidities in RA compared with other inflammatory arthritides, especially cardiovascular and metabolic disorders [16, 23]. Collectively, these studies highlight that liver disease should be recognized as part of the broader comorbidity spectrum in RA. The pathophysiological mechanisms linking RA and MASLD are likely multifactorial. Chronic systemic inflammation, insulin resistance and shared genetic risk factors contribute to hepatic steatosis [20, 24]. The role of obesity is of particular importance. Recent real-world registry data have shown that overweight and obesity substantially impact disease activity and treatment outcomes in RA patients [25]. These findings suggest that metabolic risk factors not only influence hepatic outcomes but also modify RA disease course and therapy response.

With regard to liver fibrosis, RA patients in our study exhibited higher unadjusted FIB-4 values and more frequent intermediate fibrosis risk than controls. However, these associations were no longer significant after adjustment for demographic and metabolic factors. These findings complement earlier work on the utility and limitations of non-invasive fibrosis assessment in RA patients. By additionally evaluating liver fibrosis markers, our study extends prior research that has primarily focused on hepatic steatosis or treatment-related hepatotoxicity, offering a more comprehensive picture of liver involvement in RA. Drug-related hepatotoxicity must also be considered, as methotrexate is associated with potential liver injury. The availability of non-invasive diagnostic procedures to detect methotrexate-related hepatotoxicity supports broader implementation of liver monitoring strategies in RA [22].

Our study has several strengths, including the large, well-characterized population cohort and the use of standardized MASLD definitions. Limitations include the cross-sectional design, which precludes causal inference and the lack of imaging data to confirm steatosis severity. Furthermore, we did not capture detailed RA disease activity measures or complete treatment histories.

In conclusion, our findings highlight MASLD as an underrecognized but clinically relevant comorbidity in RA. Although elevated liver fibrosis markers were observed, these were largely explained by age and metabolic factors. Integrating non-invasive liver assessment into routine RA care may therefore facilitate early detection of MASLD, particularly in patients with obesity, metabolic syndrome, or long-term methotrexate exposure. A multidisciplinary approach to RA management that incorporates hepatology and metabolic screening alongside standard rheumatologic care appears warranted.

**Table 1:** Baseline characteristics

Characteristic	Non-RA (*N* = 6,451)	RA (*N* = 187)	*P* Value
*Age—yr*	54 (49–60)	58 (54–63)	**< 0.001**
*Sex—no. (%)*			**< 0.001**
Male	2978 (46)	58 (31)	
Female	3473 (54)	129 (69)	
*HbA1c—%*	5.4 (5.3–5.6)	5.5 (5.3–5.8)	**0.004**
*Creatinine—mg/dL*	0.8 (0.7-1.0)	0.8 (0.7–0.9)	**< 0.001**
*AST—U/L*	23 (19–27)	22 (20–29)	0.20
*Gamma-GT—U/L*	21 (15–33)	22 (15–34)	0.47
*FIB-4 Score—no. (%)*			0.01
<1.30	4182 (72)	104 (63)	
1.30–2.67	1551 (27)	60 (37)	
>2.67	59 (1)	0 (0)	
*Fatty Liver Index—no. (%)*			**< 0.001**
<30	3028 (47)	74 (40)	
30 to < 60	1555 (24)	35 (19)	
≥60	1868 (29)	78 (42)	
*MASLD—no. (%)*	1842 (29)	77 (41)	**< 0.001**
*Liver stiffness—kPa*	4.5 (3.8–5.6)	4.3 (3.3–5.4)	0.26
*Total cholesterol—mg/dL*	208 (183–233)	208 (186–232)	0.97
*Triglycerides—mg/dL*	95 (70–133)	100 (72–139)	0.39
*HDL cholesterol—mg/dL*	61 (50–74)	62 (51–77)	0.15
*LDL cholesterol—mg/dL*	140 (116–164)	133 (114–158)	0.12
*hs-CRP—mg/L*	0.11 (0.06–0.23)	0.14 (0.08–0.27)	**0.010**
*BMI—no. (%)*			**< 0.001**
<30	5255 (81)	126 (67)	
≥30	1196 (19)	61 (33)	
*BMI Categories—no. (%)*			**< 0.001**
<18.5	64 (1)	3 (2)	
18.5 to 24.9	2712 (42)	60 (32)	
25.0 to 29.9	2457 (38)	63 (34)	
30.0 to 34.9	903 (14)	41 (22)	
35.0 to 39.9	228 (4)	18 (10)	
≥40	87 (1)	2 (1)	
*Type 2 diabetes—no. (%)*	223 (3)	14 (7)	**0.003**
*Hypertension—no. (%)*	1300 (20)	66 (35)	**< 0.001**
*Metabolic syndrome—no. (%)*	990 (15)	52 (28)	**< 0.001**
*Smoking status—no. (%)*			0.081
Never smoker	3000 (48)	74 (41)	
Former smoker	2159 (35)	77 (42)	
Current smoker	1051 (17)	31 (17)	
*Education level—no. (%)*			**< 0.001**
Lower education	403 (7)	30 (17)	
Medium education	4242 (69)	121 (67)	
Higher education	1470 (24)	29 (16)	

**Table 2:** Association of rheumatoid arthritis with MASLD across five Poisson regression models

	Variables Adjusted	IRR (95% CI)	*P* Value
Model I	Unadjusted	1.44 (1.21–1.72)	< 0.001
Model II	Age, sex	1.55 (1.33–1.82)	< 0.001
Model III	Age, sex, metabolic syndrome, smoking, income	1.20 (1.03–1.40)	0.019
Model IV	Model III + NSAID intake	1.15 (0.99–1.35)	0.076
Model V	SCORE2 cardiovascular risk score	1.35 (1.14–1.60)	< 0.001

### Statement of ethics

The study was approved by the Ethics Committee of the Province of Salzburg, Austria (approval no. 415-E/1521/3-2012, approval date 12 June 2012). The approval covers the Paracelsus 10,000 cohort study and subsequent secondary analyses. The study was conducted in accordance with the Declaration of Helsinki and Austrian regulatory requirements. All participants provided written informed consent prior to enrolment.

## Data Availability

The data underlying this article will be shared on reasonable request to the corresponding author.

## References

[1] Aletaha D, Neogi T, Silman AJ, Funovits J, Felson DT, Bingham CO 3rd, et al (2010) 2010 rheumatoid arthritis classification criteria: an American college of Rheumatology/European league against rheumatism collaborative initiative. Ann Rheum Dis 69(9):1580–1588. 10.1136/ard.2010.138461 10.1136/ard.2010.138461 20699241

[2] Conrad N, Verbeke G, Molenberghs G, Goetschalckx L, Callender T, Cambridge G et al (2022) Autoimmune diseases and cardiovascular risk: a population-based study on 19 autoimmune diseases and 12 cardiovascular diseases in 22 million individuals in the UK. Lancet 400(10354):733–743. 10.1016/S0140-6736(22)01349-6 36041475 10.1016/S0140-6736(22)01349-6

[3] Ausserwinkler M, Gensluckner S, Voelkerer A, Thiel J, Neumann HJ, Flamm M et al (2024) Genetic relationship between rheumatoid arthritis and cardiovascular diseases: a systematic review of Mendelian randomization studies. 10.1007/s00508-024-02392-8. Wien Klin Wochenschr 10.1007/s00508-024-02392-8 PMC12081514 39060548

[4] Park E, Bathon J (2024) Cardiovascular complications of rheumatoid arthritis. Curr Opin Rheumatol 36(3):209–216. 10.1097/bor.0000000000001004 38334476 10.1097/BOR.0000000000001004

[5] Paik JM, Golabi P, Younossi Y, Mishra A, Younossi ZM (2020) Changes in the global burden of chronic liver diseases from 2012 to 2017: the growing impact of NAFLD. Hepatology 72(5):1605–1616 32043613 10.1002/hep.31173

[6] Mantovani A, Scorletti E, Mosca A, Alisi A, Byrne CD, Targher G (2020) Complications, morbidity and mortality of nonalcoholic fatty liver disease. Metabolism 111s(154170). 10.1016/j.metabol.2020.154170 10.1016/j.metabol.2020.154170 32006558

[7] Hsu CL, Loomba R (2024) From NAFLD to MASLD: implications of the new nomenclature for preclinical and clinical research. Nat Metabolism 6(4):600–602. 10.1038/s42255-024-00985-1 10.1038/s42255-024-00985-1 PMC11262457 38383845

[8] Cotter TG, Rinella M (2020) Nonalcoholic fatty liver disease 2020: the state of the disease. Gastroenterology 158(7):1851–1864. 10.1053/j.gastro.2020.01.052 32061595 10.1053/j.gastro.2020.01.052

[9] Cho J, Lee I, Park D-H, Kwak H-B, Min K (2021) Relationships between socioeconomic status, handgrip strength, and non-alcoholic fatty liver disease in middle-aged adults. Int J Environ Res Public Health 18(4):1892 33669288 10.3390/ijerph18041892 PMC7920055

[10] Zhang S, Zhu P, Yuan J, Cheng K, Xu Q, Chen W et al (2023) Non-alcoholic fatty liver disease combined with rheumatoid arthritis exacerbates liver fibrosis by stimulating co-localization of PTRF and TLR4 in rats. Front Pharmacol 14:1149665. 10.3389/fphar.2023.1149665 37346294 10.3389/fphar.2023.1149665 PMC10279862

[11] Mori S, Arima N, Ito M, Fujiyama S, Kamo Y, Ueki Y (2018) Non-alcoholic steatohepatitis-like pattern in liver biopsy of rheumatoid arthritis patients with persistent transaminitis during low-dose methotrexate treatment. PLoS ONE 13(8):e0203084. 10.1371/journal.pone.0203084 30142184 10.1371/journal.pone.0203084 PMC6108522

[12] Frey V, Langthaler P, Raphaelis E, Ring-Dimitriou S, Kedenko L, Aigner E (2023) Paracelsus 10,000: an observational cohort study about the health status of the population of Salzburg, Austria. Rationale, objectives and study design. Paracelsus Proc Exp Med(2023 1:1–17 1033594/000000600 [CrossRef][Google Scholar]

[13] Gensluckner S, Wernly B, Koutny F, Strebinger G, Zandanell S, Stechemesser L et al (2024) Prevalence and characteristics of metabolic hyperferritinemia in a Population-Based Central-European cohort. Biomedicines 12(1). 10.3390/biomedicines12010207 10.3390/biomedicines12010207 PMC10813305 38255312

[14] Koutny F, Aigner E, Datz C, Gensluckner S, Maieron A, Mega A et al (2023) Prevalence of subclinical cardiovascular disease in patients with non-alcoholic-fatty liver disease: analysis of the paracelsus 10.000 cohort study. Med Princ Pract 32(4–5):272–280. 10.1159/000533909 37678174 10.1159/000533909 PMC10659702

[15] Dienhart C, Paulweber B, Frey VN, Iglseder B, Trinka E, Langthaler P et al (2023) Inverse association between educational status and coronary CT calcium scores: should we reflect this in our ASCVD risk assumptions? Int J Environ Res Public Health 20(12). 10.3390/ijerph20126065 10.3390/ijerph20126065 PMC10298570 37372652

[16] Ausserwinkler M, Gensluckner S, Frey V, Gostner I, Paulweber B, Trinka E et al (2025) Cerebrovascular risk in rheumatoid arthritis patients: insights from carotid artery atherosclerosis in the paracelsus 10,000 study. Rheumatol Int 45(2):33. 10.1007/s00296-024-05781-4 39825928 10.1007/s00296-024-05781-4 PMC11742769

[17] Semmler G, Wernly B, Datz C (2024) What’s in a name? New nomenclature for steatotic liver disease - to be or not to be? J Hepatol 80(2):e56–e58. 10.1016/j.jhep.2023.07.035 37567365 10.1016/j.jhep.2023.07.035

[18] Brouwers B, Rao G, Tang Y, Rodríguez Á, Glass LC, Hartman ML (2024) Incretin-based investigational therapies for the treatment of MASLD/MASH. Diabetes Res Clin Pract 211:111675. 10.1016/j.diabres.2024.111675 38636848 10.1016/j.diabres.2024.111675

[19] Ciardullo S, Mantovani A, Morieri ML, Muraca E, Invernizzi P, Perseghin G (2024) Impact of MASLD and MetALD on clinical outcomes: a meta-analysis of preliminary evidence. Liver Int 44(8):1762–1767. 10.1111/liv.15939 38597738 10.1111/liv.15939

[20] Lee Y-J, Kim KM, Ko NG, Jin M, Na JH, Park IH (2024) Effects of MAFLD defined by fatty liver index or ultrasonography on kidney function decline in the general population. Sci Rep 14(1):21189. 10.1038/s41598-024-72482-0 39261554 10.1038/s41598-024-72482-0 PMC11390887

[21] Mladenić K, Lenartić M, Marinović S, Polić B, Wensveen FM (2024) The domino effect in MASLD: the inflammatory cascade of steatohepatitis. Eur J Immunol 54(4):e2149641. 10.1002/eji.202149641 38314819 10.1002/eji.202149641

[22] Frankowski M, Świerkot J, Gomułkiewicz M, Korman L, Skoczyńska M, Starba A (2022) Usefulness of noninvasive diagnostic procedures for assessment of methotrexate hepatotoxicity in patients with rheumatoid arthritis. Rheumatol Int 42(4):631–638. 10.1007/s00296-021-05059-z 34870735 10.1007/s00296-021-05059-z PMC8940880

[23] Guła Z, Łosińska K, Kuszmiersz P, Strach M, Nowakowski J, Biedroń G et al (2024) A comparison of comorbidities and their risk factors prevalence across rheumatoid arthritis, psoriatic arthritis and axial spondyloarthritis with focus on cardiovascular diseases: data from a single center real-world cohort. Rheumatol Int 44(12):2817–2828. 10.1007/s00296-024-05740-z 39527279 10.1007/s00296-024-05740-z PMC11618134

[24] Lee ECZ, Anand VV, Razavi AC, Alebna PL, Muthiah MD, Siddiqui MS et al (2024) The global epidemic of metabolic fatty liver disease. Curr Cardiol Rep 26(4):199–210. 10.1007/s11886-024-02025-6 38376745 10.1007/s11886-024-02025-6

[25] Güler T, Yurdakul FG, Ataman Ş, Akgül Ö, Melikoğlu MA, Çapkın E et al (2025) The impact of obesity and overweight on rheumatoid arthritis patients: real-world insights from a biologic and targeted synthetic DMARDs registry. Rheumatol Int 45(9):222. 10.1007/s00296-025-05978-1 40920261 10.1007/s00296-025-05978-1

